# Supplementary material for: Direct evidence of substorm-related impulsive injections of electrons at Mercury
Source: Nat Commun. 2023 Jul 18;14:4019. doi: 10.1038/s41467-023-39565-4 (PMC10354196; doi:10.1038/s41467-023-39565-4)
Supplement: Supplementary file 1 — Supplementary Information [file 41467_2023_39565_MOESM1_ESM.pdf]

Nature Communications

Supplementary note for

Direct evidence of substorm-related impulsive injections of electrons at Mercury

Sae Aizawa[1, 2, 3]*, Yuki Harada[4], Nicolas André[1], Yoshifumi Saito[2], Stas Barabash[5], Dominique Delcourt[6], J.-A. Sauvaud[1], Alain Barthe[1], Andréi Fedorov[1], Emmanuel Penou[1], Shoichiro Yokota[7], Wataru Miyake[8], Moa Persson[1, 11], Quentin Nénon[1], Mathias Rojo[1], Yoshifumi Futaana[5], Kazushi Asamura[2], Manabu Shimoyama[5], Lina Z. Hadid[6], Dominique Fontaine[6], Bruno Katra[6], Markus Fraenz[9], Norbert Krupp[9], Shoya Matsuda[10], Go Murakami[2]

[1] IRAP, CNRS-UPS-CNES, Toulouse, France

[2] Institute of Space and Astronautical Science, Japan Aerospace Exploration Agency, Sagamihara, Japan

[3] Department of Physics, University of Pisa, Pisa, Italy

[4] Department of Geophysics, Graduate School of Science, Kyoto University, Kyoto, Japan

[5] Swedish Institute of Space Physics, Kiruna SE 98192, Sweden

[6] Laboratoire de Physique des Plasmas (LPP), CNRS-Observatoire de Paris-Sorbonne Université-Université Paris Saclay-Ecole polytechnique-Institut Polytechnique de Paris, 91120 Palaiseau, France

[7] Department of Earth and Space Science, Graduate School of Science, Osaka University, Japan

[8] Tokai University, Kanagawa, Japan

[9] Max Planck Institute for Solar System Research, Göttingen, Germany

[10] Kanazawa University, Kanazawa, Japan

[11] Graduate School of Frontier Sciences, The University of Tokyo, Kashiwa, Japan

*Corresponding author. Email: sae.aizawa@irap.omp.eu

Contents of the file:

Supplementary Method
Supplementary Fig. 1 to Fig. 5

Supplementary Note:

The data used in this file is also available in the Source Data file or Zenodo repository (https://doi.org/10.5281/zenodo.7926905) stated in Data availability section in the main paper.

Supplementary Method:

Instrumentation

The observational data used in this study are obtained by the BepiColombo mission. The BepiColombo mission consists of two spacecraft, i.e., MPO (Mercury Planetary Orbiter) led by ESA (European Space Agency) and Mio (previously called MMO, Mercury Magnetospheric Orbiter) led by JAXA (Japan Aerospace Exploration Agency). During its cruise phase, the BepiColombo mission is flying in a stacked configuration.

In this study we used data obtained by the Mercury Plasma Particle Experiment (MPPE). MPPE/MEA consists of two electron analyzers (here we use both MEA1 and MEA2) that detect low-energy electrons from 3 eV up to 26 keV. MPPE/MIA detects low-energy ions from 20 eV up to 25 keV. MPPE/ENA measures mass separated energetic neutral atoms from 10 eV to 3.3 keV. However, because of the stacked configuration during the cruise phase, some Field of View pixels also measure ions neutralized by the spacecraft sun shield.

Supplementary Figures:

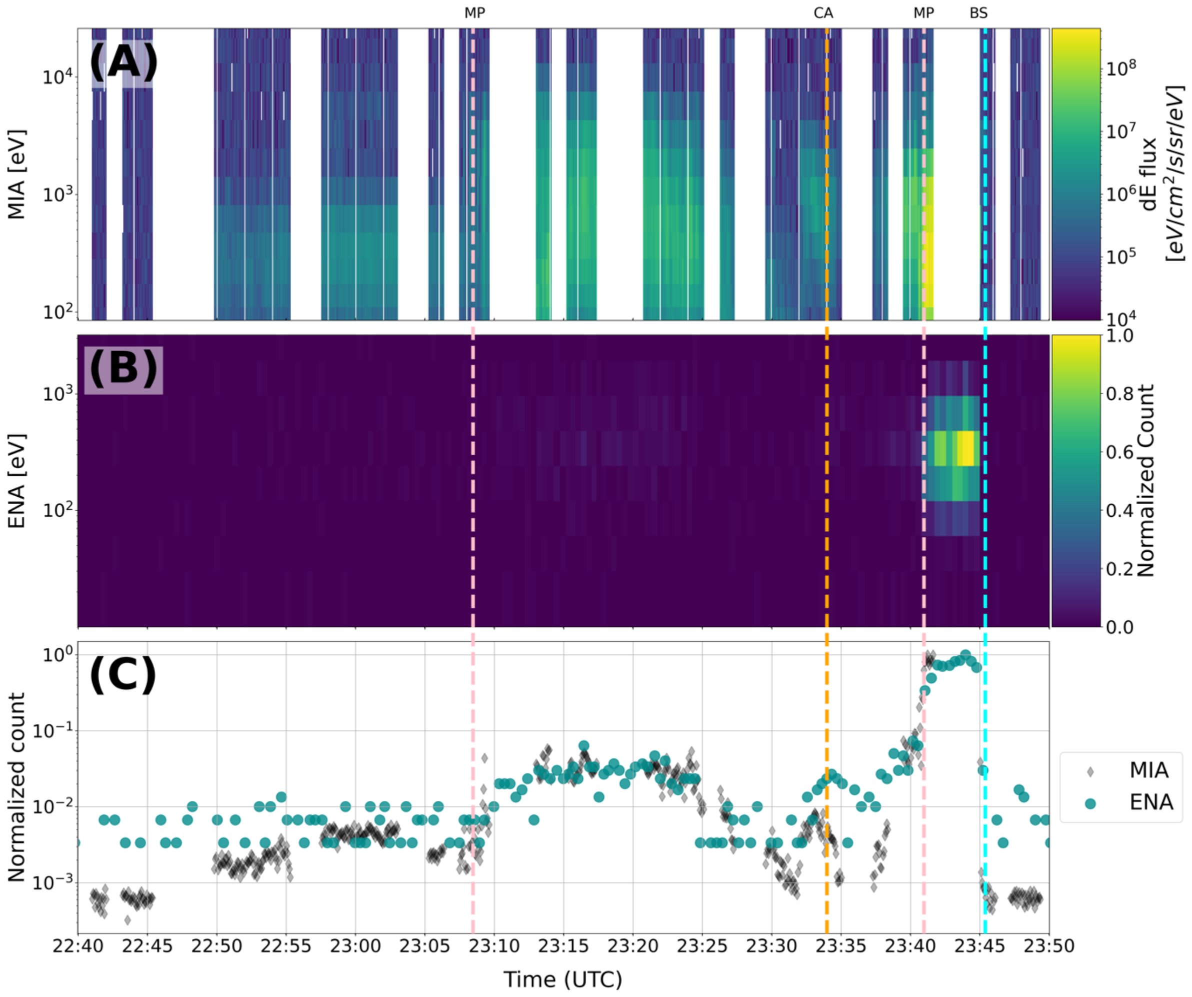

Supplementary Fig. 1. BepiColombo plasma observation obtained by Mercury Ion Analyzer (MIA) and Energetic Neutral Analyzer (ENA) during the first Mercury flyby on the 1st of October 2021. Energy-time spectrogram from (a) MIA and (b) ENA in the unit of differential energy flux, and (c) counts normalized to the maximum count observed during the time interval for both MIA and ENA. During the flyby, ENA, which is designed to measure energetic neutral atoms in orbit around Mercury, mostly observed ions neutralized when they were reflected from the Magnetospheric Orbiter Sunshield and Interface Structure. The inbound magnetopause (MP) crossing can be noticed (vertical dashed line) around 23:08:30 UTC from the observation of a gradient in the normalized counts. CA and BS indicate the closest approach and bowshock crossing.

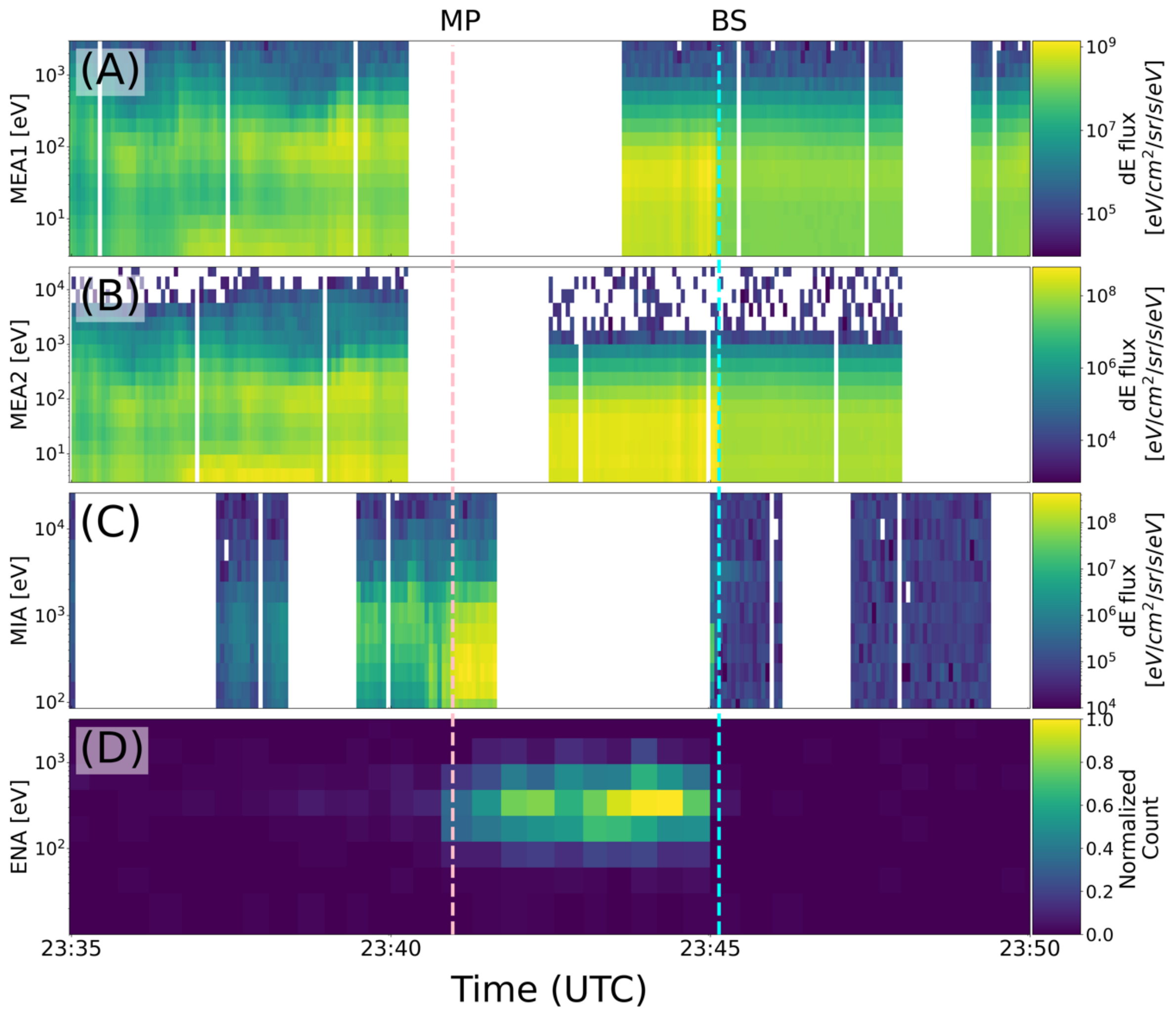

Supplementary Fig. 2. Closer look of outbound shock crossings. The format is the same as Fig. 1(B)-(E). MP and BS refer magnetopause and bowshock crossing, respectively.

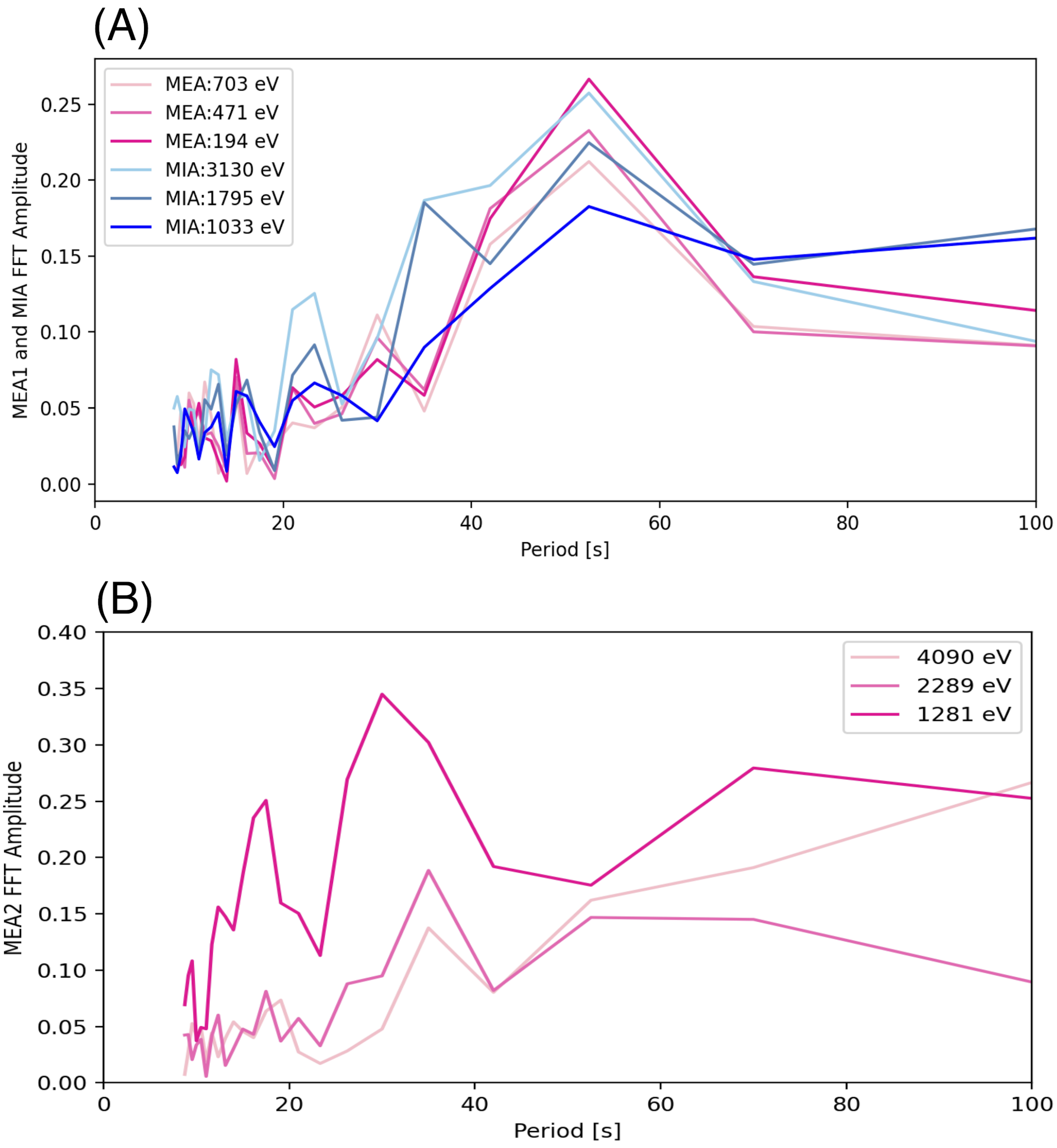

Supplementary Fig. 3. Fast Fourier Transform (FFT) applied to (A) MEA1 and MIA observations at selected energies during the time interval when the duskside ULF fluctuations are detected (23:21:30 - 23:25:00 UTC), (B) MEA2 observations at selected energies during the time interval when time-dispersed electron injections are observed (23:36:37 - 23:39:31 UTC). Data were normalized by a running average at each time interval before the FFT was applied.

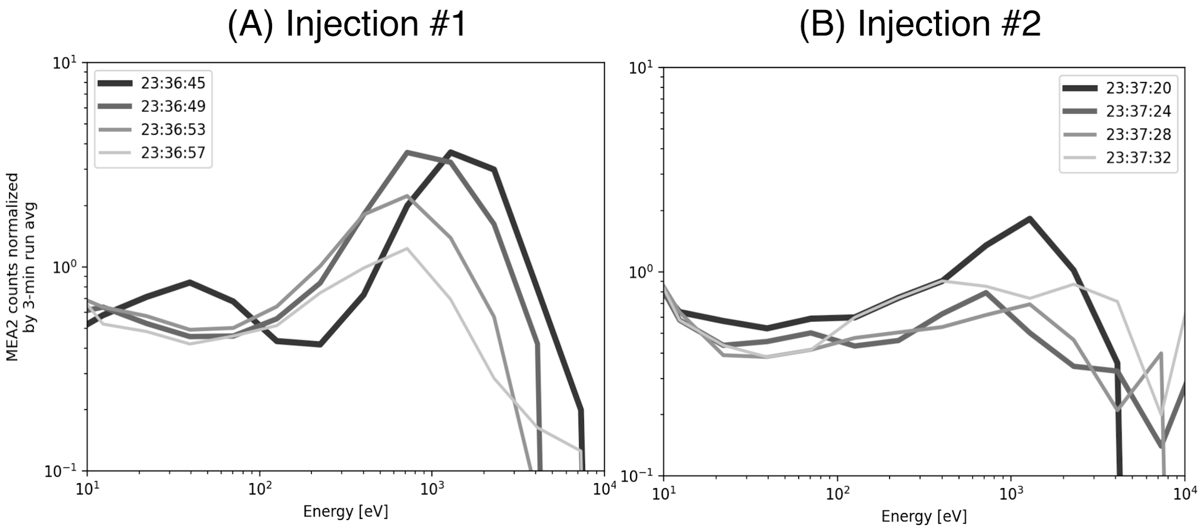

Supplementary Fig. 4. Energy distribution of MEA2 electron counts normalized by the 3-min running average (23:36:37 - 23:39:31) for electron injection #1 (A) and #2 (B). A clear energy-time dispersion can be seen above 1 keV for (A), whereas a non-dispersive electron flux enhancement is observed in (B).

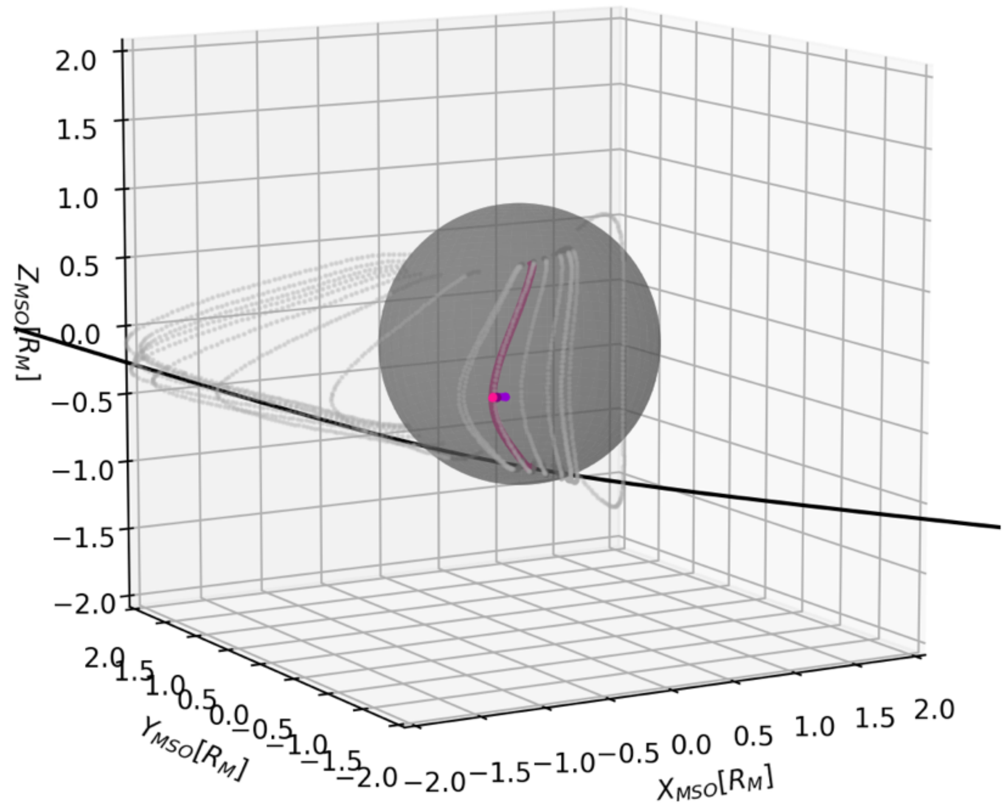

Supplementary Fig. 5 Estimated injection region by the particle tracing technique for the case of the injection #1. Grey dotted lines are the connected magnetic field lines to BepiColombo's orbit represented by solid black line. Among all grey dotted lines, pink lines and dots present electron trajectory and the estimated injection region. Injection #1 can be found where the pink lines (electron trajectory) and solid black line (BepiColombo trajectory) intersect.
